# Changing Demographics of Home Mechanical Ventilation Setups Following Acute Hypercapnic Respiratory Failure

**DOI:** 10.7759/cureus.110498

**Published:** 2026-06-08

**Authors:** Deyashini Mukherjee, Alastair Watson, Samuel Wallbanks, Amy Oakes, Giselle Pereira, Aylin Ugurlu, Rahul Mukherjee

**Affiliations:** 1 Intensive Care Medicine, University Hospitals Coventry and Warwickshire, Coventry, GBR; 2 Respiratory Medicine, Cambridge University Hospitals NHS Foundation Trust, Cambridge, GBR; 3 Respiratory Physiology, Birmingham Heartlands Hospital, Birmingham, GBR; 4 Respiratory Therapy, Birmingham Heartlands Hospital, Birmingham, GBR; 5 Respiratory Medicine, Birmingham Heartlands Hospital, Birmingham, GBR

**Keywords:** acute hypercapnic respiratory failure (ahrf), chronic obstructive pulmonary disease (copd), home mechanical ventilation (hmv), neuromuscular disease (nmd), non-invasive ventilation (niv), obesity-related respiratory failure

## Abstract

Introduction: Obesity as the primary cause of acute hypercapnic respiratory failure (AHRF) receiving acute non-invasive ventilation (NIV) has doubled within a decade. We analysed the primary diagnoses for receiving home mechanical ventilation (HMV) upon discharge following an acute NIV episode.

Methods: Data were collected from the HMV quality database of our teaching hospital between January 2014 to February 2020 (period 1: pre-COVID-19) and August 2020 to December 2021 (period 2: post-COVID-19). Adults receiving HMV upon discharge following an acute NIV episode were included. One-year and two-year survival were recorded using the National Health Service (NHS) Spine Portal. Population characteristics recorded were age, sex, primary diagnosis requiring HMV (obesity, chronic obstructive pulmonary disease (COPD), neuromuscular disease (NMD), and chest wall deformity), and concomitant home oxygen use alongside HMV. The chi-squared test was performed to assess the significance of variations between period 1 and period 2.

Results: A total of 354 patients (298 in period 1 and 56 in period 2) received HMV upon discharge following an acute NIV episode: one-year survival was 280/354 (79%), and two-year survival was 227/354 (64%). There was no significant difference between age, sex, and home oxygen use. There was a significant increase in the number of patients receiving HMV for obesity-related respiratory failure (ORRF) (113/300 (37.7%) in period 1 vs. 30/56 (53.6%) in period 2; p=0.025). There was no difference in one-year and two-year survival within each of the diagnostic categories across the two periods. As regards two-year survival, COPD had the lowest (45/103, 44%), and NMD had the highest (55/71, 77%).

Discussion: There has been a significant rise in HMV utilisation due to obesity following an episode of AHRF. This is in keeping with the rise in obesity in the population. Further work is needed to confirm whether this trend is true several years post-COVID-19, to look at the effect of comorbidities on survival in respiratory failure, and to study respiratory failure because of combined COPD and obesity.

Conclusion: In this single-centre retrospective observational study, there is a rise in HMV setups for ORRF following hospital admission for AHRF. Further multicentre work is needed to assess the significance of this trend and the impact of associated comorbidities on survival.

## Introduction

The Eurovent study assessed the use and indications of home mechanical ventilation (HMV) in Europe [1]. Since then, there have been significant changes in demographics, for example, in smoking and obesity prevalence, as well as the widening of the scientific evidence base of HMV. There isn't a specific UK home ventilation registry developed yet; however, the British Thoracic Society Model of Care does indicate that services are expected to enter information onto a national registry when it is developed [2]. While the national registry develops, we set out to look for any emerging temporal trends in the indications and demographics of HMV usage from our own quality database.

There are many established indications for HMV. In chronic obstructive pulmonary disease (COPD), following an episode of acute hypercapnic respiratory failure (AHRF), HMV results in fewer exacerbations and delays hospital readmission [3,4]. Murphy et al. showed that in patients with persistent hypercapnia (PaCO2 >53 mmHg) 2-4 weeks post-AHRF due to the acute exacerbation of COPD, those who were randomised to HMV and home oxygen vs. those randomised to home oxygen only had a significant reduction in time to readmission and death within 12 months of discharge from the hospital [4].

Obesity is an important determinant of respiratory health. Some of the symptoms commonly associated with obesity include wheeze, dyspnoea, and orthopnoea. There are many physiological effects of obesity on the respiratory system. Accumulation of fat in the mediastinum and the abdominal cavities significantly alters the mechanical properties of the lungs and chest wall, and this contributes to changes in the normal physiology and function of the lungs [5]. There is a drop in expiratory reserve volume, functional residual capacity (FRC), lung compliance, and respiratory muscle strength. Both the forced expiratory volume in one second (FEV1) and forced vital capacity (FVC) drop in obesity; however, the FEV1:FVC ratio remains the same [5]. There is an increase in airway resistance, airway flow limitation, alveolar closing volume, and ventilation-perfusion mismatch [5]. Obesity is also associated with higher levels of circulating adipokines and inflammatory cytokines, sleep-disordered breathing, and impaired respiratory drive [5]. In obesity-related respiratory failure (ORRF), HMV has led to an improvement in survival from 5% to 32%, health-related quality of life, sleep-disordered breathing, and functional parameters like FRC, FEV1, and six-minute walk test [3,6]. HMV has also been associated with a significant decrease in daytime PaCO2 [6].

The benefits of HMV in neuromuscular disease (NMD) are multifactorial, which include reduction in the work of breathing, improved neural respiratory drive, changes in respiratory mechanics, and improved sleep architecture [3,7]. HMV has also been shown to prevent nocturnal hypoventilation, hypercapnic ventilatory response, and gas exchange during daytime [3,7]. The use of HMV in these patients improves pulmonary function by increased recruitment of atelectatic zones, increased pulmonary distensibility, and improved ventilation-perfusion ratios [3,7]. HMV is one of the most important pillars of therapy in these patients, which has been shown to improve survival and quality of life [3,7]. In those with respiratory failure secondary to chest wall deformity (CWD), HMV has shown promising results with improved mortality. Much like in post-acute exacerbation of COPD, HMV has been shown to reduce hospital admission and the number of exacerbations, as well as improve gas exchange (reduction in daytime pCO2 and increase in pO2 levels) in this group [3,8].

We have previously shown that obesity as the primary cause of AHRF had more than doubled between 2004-2005 (11 out of 154 total admissions) and 2009-2010 (25 out of 160 total admissions) [9]. The length of stay for obese patients in both cohorts was significantly longer (average of 20.6 days in 2004-2005 and 19.9 days in 2009-2010) than their non-obese counterparts (8.9 days) [9]. This demonstrated a higher resource utilisation leading to higher healthcare expenditure for patients with ORRF.

Given the epidemic of obesity, we wanted to survey the demographics of the population who were using our HMV service. Our primary objective was to assess if the proportion of ORRF had increased in line with the rise in obesity as compared to the other aetiologies of respiratory failure necessitating HMV following an acute hospital admission for AHRF. Our secondary objective was to examine the one-year and two-year survival within each of the diagnostic categories across the two periods.

This article abstract was presented as an oral presentation at the British Thoracic Society Winter Meeting 2024 in London on November 27, 2024.

## Materials and methods

Data were collected from the HMV quality database of Heartlands Hospital, Birmingham, UK, part of the University Hospitals Birmingham NHS Foundation Trust, between January 2014 to February 2020 (period 1) and August 2020 to December 2021 (period 2) to exclude the first peak of the COVID-19 pandemic when the HMV service was not fully functional. We included patients who were established on HMV following an acute episode of non-invasive ventilation (NIV). We used the National Health Service (NHS) England Digital Spine Portal to get survival data. In terms of population characteristics, we included age, sex, aetiology, or diagnostic categories of respiratory failure necessitating HMV and concomitant home oxygen use.

The diagnostic categories were defined as follows: Obesity was defined as a body mass index (BMI) of ≥30 kg/m^2^. COPD was defined as a forced expiratory ratio (FEV1/FVC) of <70%. Where the primary indication was multifactorial, ORRF was identified as the predominant cause when (a) COPD, even if present, was not severe enough to cause respiratory failure (FEV1 > 50% predicted), (b) there was no current or prior diagnosis of neuromuscular weakness, and (c) BMI was >40 kg/m^2^. NMD was deemed to be the predominant cause of AHRF only in patients with prior or a new diagnosis (e.g., motor neuron disease) on that admission, never in retrospect. CWD was deemed to be the predominant cause of AHRF only in patients with proven scoliosis on chest radiographs and neither COPD severity (FEV1 >50% predicted) nor obesity.

The chi-squared test was used to compare the variation between the two time cohorts: period 1 (pre-COVID-19) and period 2 (post-COVID-19). Kaplan-Meier survival plots were used to demonstrate the probability of survival over time for individual patients in each aetiological (diagnostic) category.

This multidisciplinary audit was registered in the NHS Clinical Audit Registration and Management System (registration number: CARMS19085). Quality improvement activities using anonymised data for the purposes of improving patient care involving the retrospective analysis of existing data have prior approval from the Health Research Authority Confidential Advisory Group (CAG) under Regulation 5 of Section 251 of the NHS Act 2006 to collect patient-identifiable data without consent in England and Wales. Also, as this manuscript forms the reporting of audit cycles of a quality improvement project, individual patient consent is waived as per the Hastings Center Working Group Guidance published in 2007.

## Results

We analysed data from 354 patients in total: 298 patients from the period 1 cohort (pre-COVID-19 cohort, January 2014 to February 2020) and 56 patients from the period 2 cohort (post-COVID-19 cohort, August 2020 to December 2021). Overall, 33% of the whole cohort had concomitant home oxygen or long-term oxygen therapy (LTOT). There was no significant difference between age, sex, and home oxygen use between the two cohorts (Table 1).

**Table 1:** Overall demographics table of the participants COPD: chronic obstructive pulmonary disease; NMD: neuromuscular disease; pCO2: partial pressure of carbon dioxide; LTOT: long-term oxygen therapy; NIV: non-invasive ventilation; SD: standard deviation

Parameters		Measures
Males, n (%)		174 (48.9)
Age, years		59.7 (16.8)
Diagnosis at setup, n (%)	Obesity	143 (40.2)
	COPD	104 (29.2)
	NMD	71 (19.9)
	Chest wall deformity	37 (10.4)
pCO2 at setup (kPa), mean (SD)		9.9 (2.9)
Mode of setup, n (%)	Acute inpatient	251 (70.5)
	Elective inpatient	7 (2)
	Outpatient	92 (25.8)
	Home	6 (1.7)
LTOT prescribed at NIV setup, n (%)		118 (33.3)

There was a statistically significant increase in patients needing HMV for ORRF from 113/300 (37.8%) to 30/56 (53.6%) in the post-COVID-19 cohort (p=0.025). There was also a significantly decreased proportion of post-acute setups for NMD from 67/300 (22.4%) to 4/56 (7.1%) in the post-COVID-19 cohort (p=0.009). There were significantly more mechanical ventilation setups performed at home in the later time cohort, from no setups to six setups at home, comprising 6/56 (10%) of the post-COVID-19 cohort post-acute HMV setups (p=0.001; Table 2).

**Table 2:** Comparison of characteristics between the two cohorts *: statistically significant (p<0.05) X^2^: chi-squared statistic; COPD: chronic obstructive pulmonary disease; NMD: neuromuscular disease; pCO2: partial pressure of carbon dioxide; LTOT: long-term oxygen therapy; NIV: non-invasive ventilation; SD: standard deviation

Parameters		Pre-COVID-19 (n=300)	Post-COVID-19 (n=56)	X^2^	P-value
Males, n (%)		149 (50)*	25 (44.64)	-	0.569
Age, years		59.2 (17)*	62.4 (15.4)	-	>0.05
Diagnosis at setup, n (%)	Obesity	113 (37.8)	30 (53.6)	4.967	0.025*
	COPD	88 (29.4)	16 (28.6)	0.013	0.908
	NMD	67 (22.4)	4 (7.1)	-	0.009*
	Chest wall deformity	31 (10.4)	6 (10.7)	0.007	0.932
pCO2 at setup (kPa), mean (SD)		9.7 (2.9)	10.1 (3.1)	-	0.636
Mode of setup, n (%)	Acute inpatient	213 (71)	38 (67.9)	0.22	0.634
	Elective inpatient	6 (2)	1 (1.7)	-	>0.05
	Outpatient	81 (27)	11 (19.6)	1.33	0.24
	Home	0 (0)	6 (10.7)	-	<0.0001*
LTOT prescribed at HMV setup, n (%)*		97 (32.3)	21 (37.5)	0.568	0.451

The mean pCO2 before the acute NIV episode prior to HMV initiation for the whole cohort was 9.8kPa (SD: 2.9). When looking at the mean pCO2 across the different aetiologies of respiratory failure prior to HMV initiation, it was highest in those with COPD (mean: 10.9 kPa; SD: 3.0), followed by ORRF (mean: 10.2 kPa; SD: 2.6). The lowest pCO2 before the acute NIV episode prior to HMV initiation was in the NMD group (mean: 7.8 kPa; SD: 2.4), with those with chest wall deformity having a mean pCO2 of 8.9 kPa (SD: 2.9) prior to HMV initiation.

When looking at the whole cohort, COPD patients had the lowest one- and two-year survival, while NMD had the highest two-year survival. Interestingly, ORRF patients had the highest one-year survival across the whole cohort (124/143, 86.7%). When comparing the two cohorts, there was a significant decrease in one-year survival in the ORRF group in the post-COVID-19 cohort from 103/113 (91.2%) to 21/30 (70%; p=0.005). However, it is worth noting that it is still higher than the COPD group, where one-year survival went from 57/85 (65.5%) to 9/16 (56.3%) in the post-COVID-19 cohort (not statistically significant; Table 3).

**Table 3:** Comparison of one-year and two-year survival between the two cohorts COPD: chronic obstructive pulmonary disease; NMD: neuromuscular disease; X^2^: chi-squared statistic

		Pre-COVID-19 (n=298)		Post-COVID-19 (n=56)			
Parameters		1-year survival (n, %)	2-year survival (n, %)	1-year survival (n, %)	2-year survival (n, %)	1-year Χ^2^	2-year Χ^2^
Whole cohort		241 (80.9)	197 (66.1)	39 (69.6)	30 (53.6)	0.072	0.094
Diagnosis at setup, n (%)	Obesity	103 of 113 (91.2)	85 of 113 (75.2)	21 of 30 (70)	17 of 30 (56.7)	0.005	0.068
	COPD	57 of 87 (65.5)	40 of 87 (46)	9 of 16 (56.3)	5 of 16 (31.3)	0.573	0.412
	NMD	56 of 67 (83.5)	52 of 67 (77.6)	3 of 4 (75)	3 of 4 (75)	0.532	>0.05
	Chest wall deformity	25 of 31 (80.6)	20 of 31 (64.5)	6 of 6 (100)	5 of 6 (83.3)	0.562	0.641

**Figure 1:**
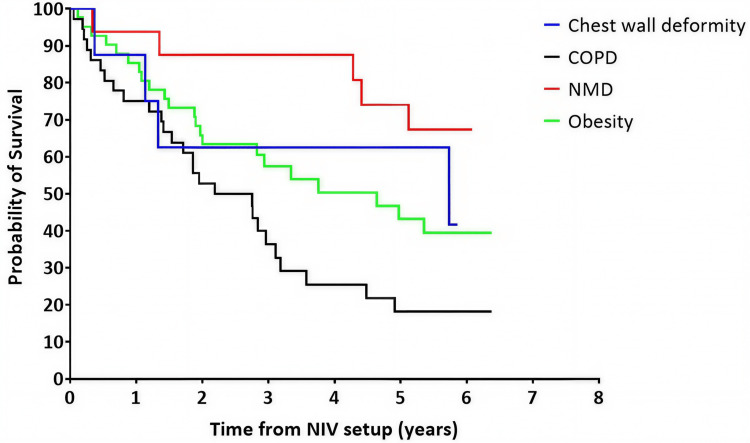
Kaplan-Meier graph of survival across the four diagnoses for cohort 1 COPD: chronic obstructive pulmonary disease; NMD: neuromuscular disease; NIV: non-invasive ventilation

The Kaplan-Meier survival analysis of the pre-COVID-19 cohort (Figure 1) shows that COPD had the lowest survival over time, followed by ORRF, while NMD had the highest survival over time.

## Discussion

We saw a significant rise in ORRF, which is in keeping with our hypothesis, mirroring the rise in obesity. ORRF is an established obesity-related complication (ORC). However, there were poorer one- and two-year survival in the obese population from the post-COVID-19 cohort, of which the one-year survival was significantly lower. This could be due to higher mortality and morbidity from the COVID-19 pandemic in the obese population for many reasons. Firstly, there is dysregulation of immunity due to higher circulating levels of pro-inflammatory cytokines such as interleukin-6 and tumour necrosis factor-alpha with lower circulating anti-inflammatory adipokine and adiponectin [10-12]. This would predispose them to more infections and COVID-19 pneumonitis itself, which would have compounded the respiratory failure from ORRF alone. Secondly, there are several cardiorespiratory and metabolic complications such as diabetes, heart failure, and chronic kidney disease that will contribute to all-cause mortality. Increased adiposity leads to endothelial dysfunction by activating several pathological biochemical cascades, including the activation of the renin-angiotensin system, activation of the procoagulant/hypercoagulation pathway, insulin resistance, oxidative stress, and platelet dysfunction [10,13-17]. A systematic review/meta-analysis found that obese patients were 1.5 times more likely to have poor outcomes due to COVID-19 and 1.09 times more likely to die [10]. Our findings support this correlation and could explain the poor survival in the obese cohort. Further analysis is needed to see how many of those with ORRF in our second time cohort also had COVID-19 and any associated acute complications such as sepsis or acute kidney injury.

**Figure 2:**
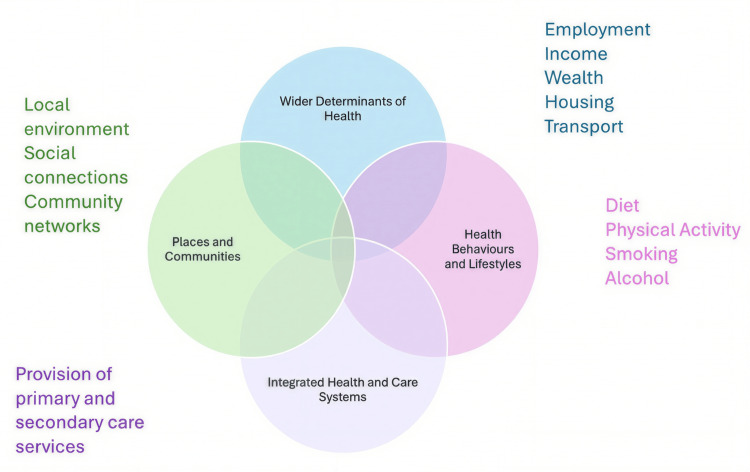
Venn diagram showing the interplay of factors contributing to the rise in obesity Image adapted from the King's Fund Tackling Obesity Report 2021 [18] and created by Deyashini Mukherjee using Microsoft PowerPoint (Microsoft Corporation, Redmond, Washington, United States)

It is well established that the prevalence of obesity is on the rise globally. Focusing on England, we can consider the contributory factors displayed in the Venn diagram (see Figure 2) [18].

Socioeconomic factors such as deprivation status, lifestyle factors, access to healthcare services, and the localities where people live all contribute to obesity [18]. While the proportion of those who are overweight or obese has steadily been climbing since 1993, the proportion of those who are obese has nearly doubled in that time from 14.9% to 28% [18,19]. Between the ages of 45 and 74, at least 30% of the population was obese [19]. When looking at men versus women across all age groups, more men were overweight or obese than women, although the proportion of obesity was comparable for men and women in most age groups [19]. Data from the Obesity Health Improvement and Disparities (OHID) office showed that among people with a disability, 73% were overweight or obese as compared to 61% in those with no disability. OHID data also showed that those of Black ethnicities had higher rates of excess weight [19]. Similarly, there was a 12% increase in rates of excess weight in people with no qualifications compared to those with a higher education level [19]. There was a 14% difference in the proportion of overweight or obese between the least and most deprived deciles, with 72% of people living in the most deprived areas falling in the overweight or obese category [19]. Comparing 2014 with 2019, there is a general rise in the proportion of obesity between the least and most deprived quintiles in men and women, going up from 2% in 2014 to 8% in 2019 for men and from 11% to 17% for women [19]. Our teaching hospital is located in Birmingham in the West Midlands, where the prevalence of overweight or obese people is 60-65% [19].

Obesity has a big effect on service development in the NHS as the costs of treating obese morbidities, such as ORRF, continue to rise [18,19]. In 2014/2015, the NHS spent £6.1 billion on treating obesity-related ill health, and this is forecast to rise to £9.7 billion per year by 2050 [19]. In 2019/2020, there were more than one million hospital admissions linked to obesity in England, an increase of 17% on the previous year [19]. Higher BMI is associated with an increase in baseline ORC prevalence and a greater increase in ORC prevalence over time [20,21]. Examples of ORCs include hypertension, type 2 diabetes mellitus, atherosclerotic cardiovascular disease including ischaemic heart disease, cerebrovascular disease, and peripheral arterial disease, obstructive sleep apnoea, heart failure, and chronic kidney disease [20,21]. Relative increases in the prevalence of individual ORCs and multimorbidity were generally greater for those with obesity relative to overweight, including for the three most common ORCs at baseline: hypertension, type 2 diabetes mellitus, and atherosclerotic cardiovascular disease [20,21].

Our results show that overall, one-year, and two-year survival in both time cohorts remains poorest in the COPD cohort, while the NMD patients had the highest survival rate across both time cohorts. This is in keeping with past literature. The Eurovent survey had shown that lung users had been on ventilation for the shortest time and neuromuscular users the longest [1]. This supports previous evidence, measured by the probability of continuing ventilation, indicating a relatively worse prognosis for lung users on long-term ventilation [1].

Several studies from around the world, including the United Kingdom and Spain, showed a temporary decrease in acute COPD exacerbation rates in the immediate post-COVID-19 period [22,23]. This may be attributed to social distancing, improved hand hygiene, and stricter adherence to infection prevention guidelines due to a perceived sense of vulnerability among COPD patients and better hand hygiene among other infection prevention factors [22,23]. There was no significant change in mortality, as seen in our cohorts.

Our findings are in keeping with the UK national respiratory support unit pilot audit 2022, which shows that ORRF is a rising cause of requiring acute NIV [24], although the post-acute (post-hospital admission) HMV setups were not studied in the national pilot. This work specifically looks at post-acute HMV setups, which reflect the same trend.

A major limitation of this study is the significantly smaller cohort in 2020-2021. This may skew the significance of the results. We are continuing to study the trends in those necessitating HMV following an episode of AHRF since December 2021. Additionally, this data only looks at patients in one hospital; therefore, the proportion of ORRF may be overrepresented given the demographics of the region the hospital caters to. The development of the national home ventilation registry will be beneficial in studying the trends in HMV utilisation across the country. The retrospective observational design of the study limits causal inference. Further subgroup analysis of demographic characteristics such as age ranges by deciles, socioeconomic factors, and ethnicity could be considered to assess whether this had any impact on the results. The presence of comorbidities such as hypertension, diabetes mellitus, ischaemic heart disease, and arrhythmias may have had an impact on the results.

Going forward, we need to do more multicentre work to establish a higher proportion of HMV utilisation due to ORRF. We also need to consider that respiratory failure is complex and can be attributed to overlapping aetiologies; for example, ORRF and COPD can co-exist and have an additive effect on the degree of respiratory failure. These overlapping diagnoses need to be studied further. We need to consider acute pathologies that may have compounded the respiratory failure, such as sepsis or acute kidney injury. Lastly, we need to consider the effect of other comorbidities on the outcomes, such as hypertension, heart failure, arrhythmias, diabetes, osteoarthritis, and depression, and use a comorbidity profiling index such as the Charlson Comorbidity Index or Adult Comorbidity Evaluation-27 (ACE-27). This could help determine the independent effect of these conditions on the long-term survival of these patients receiving HMV. Obesity is perhaps the biggest danger to the future health of all of us. We need to institute targeted efforts to prevent BMI progression and ORC development to reduce their burden on patients and healthcare systems. We need to streamline HMV pathways and enable the earlier detection of patients who would benefit from HMV through surveillance for respiratory failure and gain further insights into the natural history of respiratory failure through novel tools such as telemonitoring.

## Conclusions

We can see a trend towards more HMV setups due to ORRF in this single-centre study in a metropolitan hospital in central England, UK, which is in keeping with the overall rise in obesity across the country. The development of a UK-wide HMV national registry will help to study the trends and indications for HMV setups and see if there are geographical variations. Further work is needed to study the effect of overlapping respiratory pathologies and co-existing significant comorbidities, particularly cardiac, metabolic, and other ORCs, that may affect survival outcomes in patients utilising HMV following acute admission for AHRF.

## References

[1] Lloyd-Owen SJ, Donaldson GC, Ambrosino N (2005). Patterns of home mechanical ventilation use in Europe: results from the Eurovent survey. Eur Respir J.

[2] Messer B, Allen M, Armstrong A (2024). Model of Care for Complex Home Mechanical Ventilation. British Thoracic Society Reports.

[3] Marwah V, Dhar R, Choudhary R, Elliot M (2022). Domiciliary noninvasive ventilation for chronic respiratory diseases. Med J Armed Forces India.

[4] Murphy PB, Rehal S, Arbane G (2017). Effect of home noninvasive ventilation with oxygen therapy vs oxygen therapy alone on hospital readmission or death after an acute COPD exacerbation: a randomized clinical trial. JAMA.

[5] Dixon AE, Peters U (2018). The effect of obesity on lung function. Expert Rev Respir Med.

[6] Borel JC, Burel B, Tamisier R, Dias-Domingos S, Baguet JP, Levy P, Pepin JL (2013). Comorbidities and mortality in hypercapnic obese under domiciliary noninvasive ventilation. PLoS One.

[7] Sahni AS, Wolfe L (2018). Respiratory care in neuromuscular diseases. Respir Care.

[8] Buyse B, Meersseman W, Demedts M (2003). Treatment of chronic respiratory failure in kyphoscoliosis: oxygen or ventilation?. Eur Respir J.

[9] Thippanna CM, Thomas A, Tosh W, Chakraborty B, Beauchamp B, Banerjee D, Mukherjee R (2010). Effect of obesity in patients admitted to non invasive ventilation (NIV) unit with acute hypercapnic respiratory failure (AHRF). Thorax.

[10] Singh R, Rathore SS, Khan H (2022). Association of obesity with COVID-19 severity and mortality: an updated systemic review, meta-analysis, and meta-regression. Front Endocrinol (Lausanne).

[11] Kwaifa IK, Bahari H, Yong YK, Noor SM (2020). Endothelial dysfunction in obesity-induced inflammation: molecular mechanisms and clinical implications. Biomolecules.

[12] Daryabor G, Kabelitz D, Kalantar K (2019). An update on immune dysregulation in obesity-related insulin resistance. Scand J Immunol.

[13] Cabandugama PK, Gardner MJ, Sowers JR (2017). The renin angiotensin aldosterone system in obesity and hypertension: roles in the cardiorenal metabolic syndrome. Med Clin North Am.

[14] Blokhin IO, Lentz SR (2013). Mechanisms of thrombosis in obesity. Curr Opin Hematol.

[15] Prieto D, Contreras C, Sánchez A (2014). Endothelial dysfunction, obesity and insulin resistance. Curr Vasc Pharmacol.

[16] Virdis A (2016). Endothelial dysfunction in obesity: role of inflammation. High Blood Press Cardiovasc Prev.

[17] Anfossi G, Russo I, Trovati M (2009). Platelet dysfunction in central obesity. Nutr Metab Cardiovasc Dis.

[18] Holmes J (2021). Tackling obesity: the role of the NHS in a whole-system approach. https://assets.kingsfund.org.uk/f/256914/x/cead3911f6/tackling_obesity_role_nhs_whole_system_approach_2021.pdf.

[19] Stiebahl S (2025). Obesity statistics. https://researchbriefings.files.parliament.uk/documents/SN03336/SN03336.pdf.

[20] Pearson-Stuttard J, Holloway S, Sommer Matthiessen K, Thompson A, Capucci S (2024). Ten-year progression of obesity-related complications in a population with overweight and obesity in the UK: a retrospective open cohort study. Diabetes Obes Metab.

[21] Pearson-Stuttard J, Holloway S, Sommer Matthiessen K, Thompson A, Capucci S (2024). Variations in healthcare costs by body mass index and obesity-related complications in a UK population: a retrospective open cohort study. Diabetes Obes Metab.

[22] Lawless M, Burgess M, Bourke S (2022). Impact of COVID-19 on hospital admissions for COPD exacerbation: lessons for future care. Medicina (Kaunas).

[23] González J, Moncusí-Moix A, Benitez ID (2021). Clinical consequences of COVID-19 lockdown in patients with COPD: results of a pre-post study in Spain. Chest.

[24] (2023). National Respiratory Support Audit. https://www.brit-thoracic.org.uk/clinical-resources/clinical-audits/respiratory-support-national-audit-2023/.

